# Posterior Superior Temporal Sulcus Responses Predict Perceived Pleasantness of Skin Stroking

**DOI:** 10.3389/fnhum.2016.00432

**Published:** 2016-09-13

**Authors:** Monika Davidovic, Emma H. Jönsson, Håkan Olausson, Malin Björnsdotter

**Affiliations:** ^1^Institute of Neuroscience and Physiology, University of GothenburgGothenburg, Sweden; ^2^Center for Social and Affective Neuroscience, Linköping UniversityLinköping, Sweden; ^3^Center for Ethics, Law and Mental Health, University of GothenburgGothenburg, Sweden

**Keywords:** brain, fMRI, socio-emotional, tactile, posterior superior temporal sulcus

## Abstract

Love and affection is expressed through a range of physically intimate gestures, including caresses. Recent studies suggest that posterior temporal lobe areas typically associated with visual processing of social cues also respond to interpersonal touch. Here, we asked whether these areas are selective to caress-like skin stroking. We collected functional magnetic resonance imaging data from 23 healthy participants and compared brain responses to skin stroking and vibration. We did not find any significant differences between stroking and vibration in the posterior temporal lobe; however, right posterior superior temporal sulcus (pSTS) responses predicted healthy participant’s perceived pleasantness of skin stroking, but not vibration. These findings link right pSTS responses to individual variability in perceived pleasantness of caress-like tactile stimuli. We speculate that the right pSTS may play a role in the translation of tactile stimuli into positively valenced, socially relevant interpersonal touch and that this system may be affected in disorders associated with impaired attachment.

## Introduction

Expressions of nurturing socio-affiliative behaviors, such as love and affection, are a cornerstone in the development of attachment. Specifically, animal research emphasizes the life-long effects of physical maternal care, including licking and grooming, on nervous system maturation and behavior (Weaver et al., 2004). Indeed, interpersonal touch plays a particularly important role in brain development across a range of species (Ardiel and Rankin, 2010), and is a primary channel mediating social bonding and secure attachment in primates across the lifespan (Harlow, 1958; Dunbar, 2010; Walker and McGlone, 2013).

The temporal lobes contain key nodes of the circuitry supporting social cognition, essential for accurate perception of social cues and healthy development of attachment (Adolphs, 2009; Vrticka and Vuilleumier, 2012; Nolte et al., 2013). The posterior superior temporal sulcus (pSTS) has been identified as particularly sensitive to visually presented social information (Yang et al., 2015). A growing number of studies suggest that the pSTS may also contain multisensory circuits (Beauchamp, 2010), including areas responding to tactile stimulation (Beauchamp et al., 2008). Given the profound role interpersonal touch plays in social interactions and attachment (McGlone et al., 2014), it seems likely that the pSTS may be selectively sensitive to socially relevant tactile information. Indeed, recent studies show that caress-like gentle skin stroking of the forearm activates the right pSTS (Gordon et al., 2013; Voos et al., 2013; Björnsdotter et al., 2014). Moreover, pSTS responses to skin stroking show sex-specific developmental effects (Björnsdotter et al., 2014), correlate inversely with autistic traits (Voos et al., 2013) and are reduced in individuals diagnosed with autism spectrum disorder (Kaiser et al., 2015). Taken together, these findings demonstrate a role of the pSTS in tactile processing, but also link temporal lobe functionality to individual variability in social behavior.

Already at the level of the skin, specific peripheral nerves in the form of small-diameter, unmyelinated C tactile (CT) afferents signal socio-affective dimensions of touch (Löken et al., 2009; Björnsdotter et al., 2010; Morrison, 2012; Ackerley et al., 2014). Contrary to large-diameter myelinated mechanoreceptive (Aβ) afferents, CT nerve fibers respond particularly well to gentle, caress-like slow skin stroking (Löken et al., 2009; Ackerley et al., 2014) but poorly to rapid skin deformation such as vibrotactile stimuli (Bessou et al., 1971; Olausson et al., 2002). Physical touch mediates social bonding and attachment in primates (Harlow, 1958; Dunbar, 2010), and the CT system is posited to play a particularly important role in the development of attachment (McGlone et al., 2014). Moreover, pure CT stimulation evokes a vaguely pleasant percept (Olausson et al., 2002), and CT afferent firing frequency correlates with perceived pleasantness of skin stroking (Löken et al., 2009; Ackerley et al., 2014).

Consistent with a socio-affective role of the CT system, previous studies suggest that posterior temporal lobe regions responsive to touch may be selective to the type of skin stroking that vigorously activates CT afferents. Stroking of the hairy skin of the arm elicits more activity than that of the glabrous skin of the palm (Gordon et al., 2013; Björnsdotter et al., 2014) which is not innervated by CT afferents (Björnsdotter et al., 2010). Also, the STS is more responsive to slow than fast skin strokes (Voos et al., 2013). However, the extent to which the STS is selective to CT targeted stimulation is unclear; specifically, the STS is known to respond to skin vibration (Beauchamp et al., 2008), which poorly activates CT afferents (Bessou et al., 1971; Olausson et al., 2002). In the current study, we therefore used functional magnetic resonance imaging (fMRI) to contrast brain responses to skin stroking and vibration in healthy participants.

In addition to having dissimilar effects on Aβ and CT afferents, skin vibration is an artificial type of touch that is not associated with social processes. The right pSTS is selectively responsive to point-light displays of biological motion, including depictions of human movements compared to randomly moving dots (Grossman and Blake, 2002). Similar to such displays, purposeful gentle skin stroking is an inherently social stimulus, in contrast to vibration, which can be considered an unnatural, non-social type of touch. Consistent with previous studies showing selective activations of the posterior temporal lobe to socially relevant touch (Gordon et al., 2013; Voos et al., 2013; Björnsdotter et al., 2014), we hypothesized that the pSTS would respond more to skin stroking than to skin vibration. Superior temporal cortex activity is also modulated by a wide array of factors related to individual variability, such as social impairment (Kaiser et al., 2010), autism diagnosis (Björnsdotter et al., 2016), perceived level of animacy (Kuzmanovic et al., 2014), and plasma oxytocin (Lancaster et al., 2015). Specifically, temporal responses to skin stroking are affected by age and gender (Björnsdotter et al., 2014), autistic traits (Voos et al., 2013), and autism diagnosis (Kaiser et al., 2015). Here, we asked whether pSTS activity may also be modulated by individual percepts of the affective quality of tactile stimulation. In line with previous studies of the CT system (Olausson et al., 2002; Löken et al., 2009; Ackerley et al., 2014), we therefore asked the participants to rate the perceived pleasantness of the tactile stimuli. Consistent with the role of the pSTS in processing socially relevant touch (Gordon et al., 2013; Voos et al., 2013; Björnsdotter et al., 2014), we hypothesized that pleasantness ratings of skin stroking, but not vibration, would correlate with pSTS responses.

## Materials and Methods

### Participants

Participants were recruited through university advertisements. Twenty-three subjects (11 males, mean age 25 years, range 19–38 years) participated in the study. All subjects were right handed as assessed through the Edinburgh Handedness Inventory, and healthy. Ethical approval was obtained by the ethics board of the Gothenburg University, and the study was performed in line with the guidelines of the Declaration of Helsinki (1996). Participants were compensated with 200 Swedish crowns per hour.

### Tactile Stimuli and Experimental Protocol

A trained experimenter (author MD; female, aged 44) applied the stimuli by hand, guided by visual cues. The experimenter was invisible to the participants throughout the scanning session. Gentle skin stroking was applied by a 6-cm wide artist’s brush at a speed of 2 cm/s across a distance of 10 cm, in a proximal to distal direction on the right anterolateral surface of the thigh. Vibration (100 Hz) was delivered with a device consisting of a rectangular piece (40 mm × 12 mm × 7 mm) of balsa wood connected to a piezo-element (Piezo Systems Inc., Cambridge, MA, USA).

**FIGURE 1:**
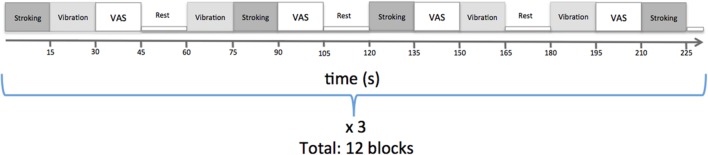
**Experimental paradigm**.

Each tactile stimulus lasted for 15 s and the stimuli were administered in a pseudo-randomized order. The order of the stimuli was visible only to the experimenter who administered the touch. Each block contained one stroking stimulus, one vibration stimulus, and a behavioral assessment (**Figure 1**). In the behavioral assessment, participants were asked to rate the perceived pleasantness of the last stimulus on a visual analog scale (VAS) presented on a screen visible for the participant through a mirror attached to the head coil. The scale was visible for 15 s, during which the participants could move a cursor using buttons. The cursor was initially placed at the center marked ‘neutral,’ and the endpoints of the VAS were ‘unpleasant’ (‘obehagligt’ in original Swedish language) and ‘pleasant’ (‘behagligt’ in original Swedish language). The visual cues and VAS scales were presented using custom designed Matlab scripts, which were synchronized with scanner data collection. Participants were instructed to focus on the screen throughout the experiment. For statistical assessment, the scale was subsequently converted to the range -5 to 5, and the average value was computed across all blocks for stroking and vibration, respectively. The scanning session comprised one run with 12 blocks, each block separated by a 15 s rest period (**Figure 1**).

### MRI Acquisition

Magnetic resonance imaging was performed on a Philips Gyroscan 3T Achieva, software release 3.2, (Philips, Eindhoven, The Netherlands). The scanner’s two channel parallel transmit was used for improved signal homogeneity over the field of view and the subject’s head was firmly supported with cushions in the head coil (32 channel SENSE, same manufacturer as the scanner). A T1-weighted scan (3D T1-TFE) was performed as anatomical reference (parameters: flip angle 8°, *TE* = 4.0 ms, *TR* = 8.4 ms, SENSE factor 2.7, TFE factor 240, 170 sagittal slices with scan resolution 1.0 mm × 1.0 mm × 1.0 mm). Functional data comprised 245 volume images of the brain (parameters: single shot gradient echo, echo planar imaging with flip angle 90°, *TE* = 35 ms, *TR* = 3000 ms, SENSE factor 1.8, 40 axial slices without slice gap and with scan resolution 2.8 mm × 2.8 mm × 2.8 mm), acquired after four discarded dummy scans.

### Data Pre-processing

All spatial preprocessing and statistical analyses of anatomical and functional images were performed using SPM8^1^. The anatomical images were segmented into gray matter, white matter, and cerebrospinal fluid images. The gray matter images were used to determine the 12-parameter affine transformation onto the standard stereotactic MNI (Montreal Neurological Institute) space. Functional data preprocessing included slice time correction, realignment to the first volume of the first run (using a 6-degree rigid spatial transform), co-registration to anatomical images, transformation to MNI space using the parameters obtained from transformation of gray matter images, resampling to 2 mm × 2 mm × 2 mm voxels and smoothing with a 6-mm full width at half maximum Gaussian kernel. In addition, motion artifacts were examined using the Artifact Detection Toolbox (ART)^2^. Volumes in which global signal deviated more than two standard deviations (SDs) from the mean signal or in which the difference in motion between two neighboring volumes exceeded 1 mm (across rotational or translation directions) were marked as outlier volumes. Smoothed functional images were band pass-filtered with a 128 s high pass filter.

### General Linear Modeling

Four regressors corresponding to the four conditions (stroking, vibration, VAS rating for stroking, and VAS rating for vibration) were modeled using a boxcar function with 1 during the 15 s stimulus conditions and 0 otherwise, convolved with a canonical hemodynamic response function. The design matrix also included motion parameters and outlier volumes as regressors of no interest. The mean number of outliers per participant was 1.91 (*SD* = 2.66, range = 0–8). There was no significant difference in number of outliers between stroking and vibration (*p* = 0.3). Parameter estimates of blood-oxygen level dependent (BOLD) responses (β-values) were calculated for each tactile condition (stroking and vibration) and for the difference between conditions. These were passed to a second level mixed effect group analysis. As there are sex-specific age-effects on brain responses to tactile stimuli (Björnsdotter et al., 2014), we included gender and age as covariates.

We examined the main effect for each of the two tactile stimuli, as well as the contrast between them. The resulting statistical maps were thresholded at whole-brain family-wise error (FWE) corrected *p* < 0.05 and cluster size > 5.

### Region-of-Interest (ROI) Analyses

We conducted ROI analyses in the right pSTS, with Talairach space coordinates reported in the seminal paper on temporal processing of CT targeted touch ([57, -55, 13]; Gordon et al., 2013). Here, we converted the coordinates to MNI space in GingerAle (Laird et al., 2011), resulting in [55, -53, 15], and constructed the right pSTS ROI as a sphere with radius 8 mm centered on this coordinate. Within this ROI, we applied small volume correction (SVC) for multiple comparisons at pFWE < 0.05.

### Correlation Analyses

We assessed brain-behavior links in the form of correlations between brain responses and participants’ pleasantness ratings. Specifically, we assessed the correlations between β-values for each tactile condition and the respective pleasantness ratings. First, we used the MarsBaR toolbox^3^ to extract average β-values for stroking and vibration from the right pSTS ROI and computed the correlations between these values and the pleasantness ratings. Second, we conducted an exploratory group-level, whole brain, random effects analysis to examine voxel-wise correlations between ratings and brain responses to stroking and vibration, respectively, including age and gender as a covariates. The results were reported at an uncorrected *p* < 0.001 and cluster size > 5.

## Results

### Behavioral Ratings

**FIGURE 2:**
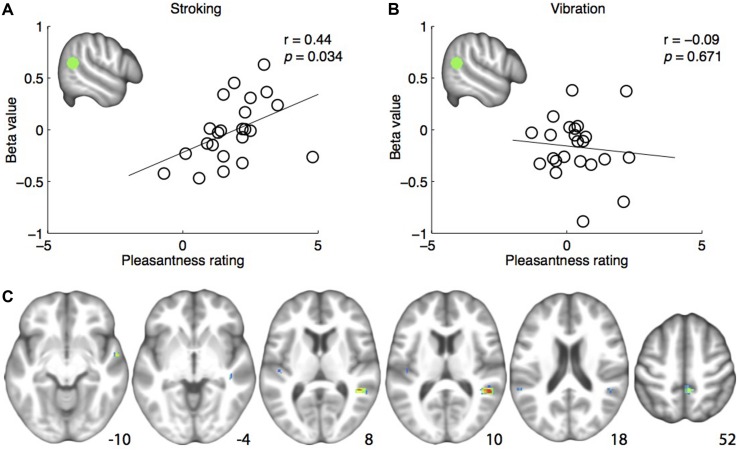
**(A)**Positive correlation between pleasantness ratings and brain responses to skin stroking in the pre-defined right posterior superior temporal sulcus region-of-interest (rpSTS) (indicated in green). **(B)** No correlation between pleasantness ratings and brain responses to skin vibration in the rpSTS. **(C)** Whole-brain correlations between brain responses to skin stroking and pleasantness ratings, shown at *p* < 0.001. Coordinates are indicated in Montreal Neurological Institute (MNI) space.

Skin stroking ratings ranged from neutral to very pleasant (**Figure 2A**), and vibration ratings ranged from slightly unpleasant to slightly pleasant (**Figure 2B**). The group mean pleasantness ratings were 1.9 (*SD* = 1.1) and 0.4 (*SD* = 0.9) for skin stroking and vibration, respectively. Within subjects, the SDs of the pleasantness ratings were small, ranging from 0.1 to 1.8 for skin stroking and 0.1 to 1.1 for vibration. Participants experienced stroking as more pleasant than vibration (paired samples *t*-test, *p* < 0.001). Stroking, but not vibration, was rated significantly higher than 0 (‘neutral’; *p* < 0.001 and *p* = 0.08, for stroking and vibration, respectively).

### Neuroimaging

#### Main Effects of Tactile Stimuli

**FIGURE 3:**
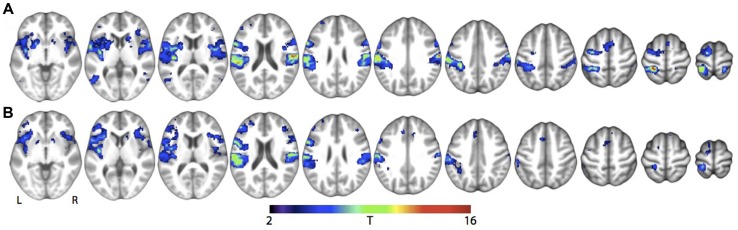
**Main effect of tactile stimulation vs. rest for (A) Skin stroking and (B) Skin vibration.** Results are shown at whole-brain *p* < 0.001, uncorrected for multiple comparisons.

For the main effects of skin stroking and vibration, respectively, we found activations in a range of somatosensory areas previously associated with tactile stimulation, such as bilateral secondary somatosensory cortex and contralateral primary somatosensory cortex (**Figure 3**; **Table 1**). However, we did not find any significant activation of the pSTS for skin stroking, even at the lower threshold of *p* < 0.001 or within the right pSTS ROI.

**Table 1:** Results from the whole brain analyses.

	Region		Peak *z*-value	*x*	*y*	*Z*	Nr voxels
Stroking	Left	Postcentral gyrus	7.30	-24	-42	62	1548
	Right	Postcentral gyrus	6.80	56	-18	18	503
	Right	Insula	6.11	40	-2	14	48
	Left	Superior frontal gyrus	5.91	-14	-10	72	14
	Left	Inferior frontal gyrus	5.76	-56	8	22	89
	Left	Precentral gyrus	5.43	-34	-10	56	19
	Left	Lentiform nucleus	5.34	-20	6	-6	8
	Right	Inferior frontal gyrus	5.14	60	10	30	5
	Right	Superior temporal gyrus	5.09	60	6	2	5

Vibration	Left	Postcentral gyrus	6.60	-60	-18	16	563
	Left	Inferior frontal gyrus	5.85	-56	8	24	112
	Right	Postcentral gyrus	5.84	50	-16	18	142
	Left	Insula	5.71	-40	-12	4	52
	Left	Undefined	5.36	-54	10	4	15
	Left	Postcentral gyrus	5.25	-22	-40	68	13
	Left	Inferior frontal gyrus	5.23	-40	16	-4	21

Stroking>	Left	Postcentral gyrus	6.13	-54	-22	36	132
Vibration	Left	Inferior frontal gyrus	5.78	-58	6	30	17
	Left	Postcentral gyrus	5.65	-24	-48	68	146
	Right	Postcentral gyrus	5.63	20	-44	70	20
	Left	Precentral gyrus	5.58	-34	-10	56	9
	Right	Precentral gyrus	5.45	56	-18	36	17
	Right	Postcentral gyrus	5.44	54	-16	24	29

Stroking –	Right	Superior temporal gyrus	4.44	50	-44	10	137
Rating^1^	Right	Middle temporal gyrus	4.08	58	-4	-10	18
Correlation	Right	Paracentral lobule	4.03	6	-44	52	72
	Right	Insula	3.39	42	-28	-4	9
	Left	Superior temporal gyrus	3.35	-56	-42	18	15
	Left	Superior temporal gyrus	3.24	-44	-24	8	5

*Results are assessed at family-wise error corrected p** <** 0.05, unless otherwise specified.^1^Reported at p** <** 0.001, uncorrected for multiple comparisons.*

#### Effects of Skin Stroking vs. Vibration

We found multiple of brain regions in which stroking elicited a significantly larger response than vibration (**Table 1**). However, we found no significant differences between skin stroking and vibration in the STS, at the lower threshold of *p* < 0.001 or within the right pSTS ROI. Also, no region with significantly larger responses for vibration than skin stroking was found.

#### Correlation Analysis

The analysis examining the correlation between right pSTS ROI β-values and pleasantness ratings showed a significant positive correlation for stroking (*r* = 0.444, *p* = 0.034) (**Figure 2A**). For vibration, the correlation was not significant (*r* = -0.098, *p* = 0.657) (**Figure 2B**). Consistently, a one-tailed *t*-test on the z-transformed correlation coefficients showed that the skin stroking correlation coefficient was significantly larger than the vibration coefficient (*p* = 0.034).

The whole-brain exploratory analysis revealed a number of regions exhibiting a correlation between brain responses and pleasantness ratings for stroking (uncorrected *p* < 0.001) (**Table 1**). Of these, a cluster with a peak in the superior temporal gyrus, extending well into the STS, was the largest (**Figure 2C**). No voxels passed the threshold for vibration.

## Discussion

We examined the extent to which posterior temporal lobe responses are selective to socio-affective touch by comparing activity elicited by skin stroking and skin vibration. Contrary to our hypothesis, we found no significant differences between skin stroking and vibration. However, right pSTS responses correlated significantly with participants’ perceived pleasantness of skin stroking, but not vibration. These results suggest that right pSTS responses may indeed be selective to skin stroking, but also that pSTS activity is modulated by individual variability in perceived affective quality of touch.

Our study did not replicate previous findings of significant group level pSTS responses to skin stroking, and skin stroking did not elicit significantly more temporal lobe activity than vibration on the group level. However, the finding of a positive correlation between pleasantness ratings and pSTS responses to skin stroking, but not vibration, suggests that the lacking group level effects may be partially due to statistical issues. Specifically, we applied voxelwise correction for multiple comparisons, which was recently demonstrated to be substantially more conservative than clusterwise correction (Eklund et al., 2016) used in previous studies (Gordon et al., 2013; Voos et al., 2013; Björnsdotter et al., 2014). As such, there may have been STS effects that would have survived a less stringent initial threshold (of e.g., *p* < 0.005) with clusterwise correction for multiple comparisons, but that did not pass our least stringent voxelwise threshold (*p* < 0.001). In the current study, we did not assess the effects using less stringent criteria due to the high risk of obtaining type I errors (Eklund et al., 2016). The experimental design, in which stroking and vibration stimuli were interleaved with no interstimulus interval, is less likely to have caused the lacking effect: this design was successfully applied in a similar previous study (Morrison et al., 2011), albeit using a larger number of stimuli repetitions but also substantially fewer participants, and we identified condition differences across a large number of other brain regions previously associated with tactile stimulation at the highly conservative threshold of whole-brain pFWE < 0.05.

In light of potential statistical processing differences, our finding that pSTS responses to skin stroking, but not to vibration, correlate with pleasantness ratings is highly consistent with previous findings of a selective role of the right pSTS in processing socially relevant tactile cues (Gordon et al., 2013; Voos et al., 2013; Björnsdotter et al., 2014). Although vibrotactile stimulation may be linked to social processing – the vibration probe was manually applied by the experimenter, and vibration is becoming increasingly associated with communication through cell phones (Drouin et al., 2012) – our data did not reveal any significant effects in relation to skin vibration. However, since vibrotactile stimuli is known to activate the temporal lobe (Beauchamp et al., 2008), we speculate that paradigms specifically interrogating social processes linked to vibration, such as the behavioral response to the regular repeated buzz of a cell phone call, may detect such effects. Similarly, the current study did not attempt to dissociate social and CT-mediated touch processing, as gentle skin stroking is both the preferred CT stimulus and an inherently social type of touch. Instead, future studies using factorial designs including social/non-social and CT/non-CT mediated touch are required to establish whether the observed effects are related to the social component of touch or to the CT system.

The finding of a correlation between brain responses to skin stroking and pleasantness ratings supports the previously demonstrated link between variability in pSTS processing and social behavior. The superior temporal cortex is modulated by an astonishing variety of individual parameters, including task performance (Herrington et al., 2011), cognitive ability (Rutherford and Troje, 2012), social impairment (Kaiser et al., 2010), perceived animacy (Kuzmanovic et al., 2014), motor skills (Freitag et al., 2008), serotonin transporter genotype (Fisher et al., 2015), and plasma oxytocin (Lancaster et al., 2015). In line with these findings, we speculate that the demonstrated correlation may reflect a range of individual factors related to socio-affective sensory dimensions, rather than varying levels of peripheral input or low-level processing. For instance, early experiences such as frequency of maternal touch (Brauer et al., 2016), attachment-related stress (Nolte et al., 2013), and a range of other attachment-related processes (Vrticka and Vuilleumier, 2012) influence the functioning of social brain regions such as the STS. Since we did not assess these measures in the current study, future studies are needed to disentangle the relative contributions of such factors, as well as to identify additional brain circuits that may contribute to the coding of affective aspects of touch.

Conforming to previous research on the CT system (Löken et al., 2009; Morrison et al., 2011), we asked participants to rate the tactile experience in terms of pleasantness. The term ‘pleasant’ is not sufficiently concise to allow a precise interpretation, however; for example, interpersonal touch may feel pleasant in terms of sensory hedonics (such as ‘softness’) but unpleasant in terms of social aspects (such as ‘unfamiliarity’) (Gentsch et al., 2015). Hence, it is not clear whether the demonstrated correlation reflects a sensory-hedonic or social-affective dimension of the tactile sensation. Since the pSTS is robustly linked to social (Adolphs, 2009), rather than emotional or hedonic processing, we speculate that the observed correlation is primarily governed by a social-affective dimension.

Given the role of the pSTS in integrating sensory and social information (Yang et al., 2015), we further propose that the pSTS may play a role in the translation of neutral tactile stimuli into positively valenced, socially relevant touch. As such, this system may be affected in psychiatric conditions associated with altered social behavior and impaired attachment, including autism spectrum disorder (ASD). Consistent with this notion, just-published findings show reduced right pSTS responses to skin stroking in children with ASD (Kaiser et al., 2015). Socially relevant tactile behaviors extend well beyond skin stroking and caressing, however, including hugging, kissing, tickling, and so on; further studies are required to elucidate any generalized role of the temporal lobes across such behaviors.

## Conclusion

Our results support a role for the posterior temporal lobe in processing socio-affective dimensions of touch. Specifically, our study supports the notion that socio-affective touch may be selectively processed in the temporal lobe; however, our results also suggest that any selectivity is contingent on top-down effects related to subjectively perceived qualities of the tactile stimulation.

## Author Contributions

MD and EJ designed the study and collected data. MD and MB performed the analyses. All authors participated in the interpretation of the data. MD an MB drafted the article. All authors contributed to the final revision of the article.

## Conflict of Interest Statement

The authors declare that the research was conducted in the absence of any commercial or financial relationships that could be construed as a potential conflict of interest.

## Footnotes

1 http://www.fil.ion.ucl.ac.uk/spm/

2 http://www.nitrc.org/projects/artifact_detect/

3 http://marsbar.sourceforge.net/
